# Why individuals with trait anger and revenge motivation are more likely to engage in cyberbullying perpetration? The online disinhibition effect

**DOI:** 10.3389/fpubh.2025.1496965

**Published:** 2025-01-28

**Authors:** Jin-liang Ding, Yan Lin, I-Hua Chen

**Affiliations:** ^1^School of Humanities and Teacher Education, Wuyi University, Wuyishan, China; ^2^Fuzhou University of International Studies and Trade, Fuzhou, China; ^3^Chinese Academy of Education Big Data, Qufu Normal University, Qufu, China

**Keywords:** trait anger, cyberbullying perpetration, revenge motivation, toxic online disinhibition, benign online disinhibition, adolescents

## Abstract

**Background:**

Trait anger has been identified as a significant risk factor in cyberbullying perpetration; however, the mechanisms underlying this relationship remain underexplored. This study aims to elucidate the connection between trait anger and cyberbullying perpetration among adolescents, with a focus on the mediating role of revenge motivation and the moderating effect of online disinhibition.

**Methods:**

A sample of 1,574 Chinese adolescents (46.1% female, mean age 16.89 years, SD = 0.34) participated in the study. Participants completed measures assessing trait anger, revenge motivation, online disinhibition, and cyberbullying perpetration.

**Results:**

Revenge motivation partially mediated the association between trait anger and cyberbullying perpetration. Furthermore, the relationship between revenge motivation and cyberbullying perpetration, as well as the relationship between trait anger and cyberbullying perpetration, were moderated by online disinhibition, with a significant association observed only among adolescents exhibiting higher levels of toxic disinhibition.

**Conclusion:**

These findings extend the current understanding of cyberbullying perpetration among adolescents and offer valuable insights for intervention strategies targeting this antisocial online behavior.

## 1 Introduction

Cyberbullying perpetration, defined as intentional and harmful behavior performed by an individual or group using electronic devices, repeatedly and over time against a victim who cannot easily defend themselves (1), has emerged as a significant concern in the digital age. Research across cultures has documented the detrimental effects of cyberbullying on adolescents’ psychological well-being. While Western studies emphasize increased risks of substance use, anxiety, and depression (2, 3), research in China has identified additional culture-specific impacts, such as heightened academic pressure, family relationship strain, and unique manifestations of social withdrawal (4, 5). These cultural differences in both the expression and impact of cyberbullying highlight the importance of examining this phenomenon within specific cultural contexts.

A growing body of literature has identified trait anger (TA) as a predictive factor for cyberbullying perpetration [e.g., (6–8)]. TA, defined as a tendency to experience irritation, annoyance, and rage (9), influences how individuals experience and express anger. Studies in China have revealed how collectivist cultural values shape the expression of trait anger in cyberbullying contexts (10–12), particularly given cultural emphases on emotional restraint and harmony maintenance (13).

In addition to trait anger, the online disinhibition effect presents unique characteristics in the Chinese digital landscape. While disinhibition refers to reduced behavioral restraint and concern for self-representation (14), its manifestation in Chinese digital spaces is shaped by distinct platform governance structures. Unlike Western platforms, Chinese social networking platforms operate under a real-name system that requires users to register with their actual information (15). This creates a unique environment for examining online disinhibition, which can be classified as either benign or toxic depending on the nature of the behavior (16). Studies in Chinese contexts suggest distinctive patterns of online disinhibition influenced by this real-name system and cultural norms (17).

Although previous research has examined individual characteristics, online disinhibition, and cyberbullying perpetration separately, limited research has considered their interrelationships within specific cultural contexts, especially in environments with mandatory real-name systems. To address these research gaps, the current study examines the link between trait anger and cyberbullying perpetration within the Chinese cultural context, investigating the mediating role of revenge motivation and the moderating role of online disinhibition among Chinese adolescents.

### 1.1 The mediating role of revenge motivation

Revenge motivation refers to the intention of a victim to inflict damage, injury, discomfort, or punishment on the party deemed responsible for causing harm (18). Although revenge is not the sole motivator for cyberbullying—including social status enhancement (19), entertainment (20), or perceived peer pressure (21)—it represents a significant pathway through which trait anger may lead to cyberbullying behaviors.

The General Aggression Model (GAM) (22) provides a theoretical framework for understanding how trait anger might lead to revenge motivation. The GAM suggests that personal factors (such as personality traits) and situational factors (like cues of aggression) influence internal states, including cognition, emotion, and arousal, which in turn affect appraisal and decision processes, ultimately leading to aggressive behavior. In this context, trait anger, as a stable personality characteristic predisposing individuals to experience frequent and intense anger across situations, may consistently influence how people process social information and respond to perceived provocations.

The connection between trait anger and revenge motivation can be understood through several mechanisms. First, trait anger influences cognitive processing: individuals high in trait anger tend to exhibit hostile attribution bias, leading them to consistently interpret ambiguous situations as threatening or provocative (23). This hostile interpretation style creates a cognitive foundation for revenge motivation. Second, trait anger affects emotional reactivity: high trait-angry individuals experience more frequent and intense angry emotions in response to provocations because their amygdala has weaker functional connectivity with the contralateral orbitofrontal cortex, and they have poorer ability to regulate their emotions (24), making them more likely to consider revenge as a viable response. Third, trait anger influences behavioral tendencies: those high in trait anger show lower thresholds for aggressive responses and are more likely to endorse revenge as a legitimate solution to perceived wrongs (25). These theoretical connections are supported by empirical evidence. For example, Wilkowski et al. (26) found that individuals with high trait anger showed stronger tendencies toward retaliatory responses when provoked. This finding aligns with the concept that trait anger creates a predisposition toward revenge-seeking behavior. Additionally, studies have demonstrated that high trait-angry individuals maintain angry thoughts longer (rumination) and are more likely to develop revenge plans in response to provocations (27, 28).

Importantly, revenge-motivated cyberbullying presents a unique dynamic in terms of power relationships. While traditional cyberbullying definitions emphasize power imbalance between perpetrator and target, revenge-based cyberbullying often involves what König et al. (29) termed “power redistribution,” where victims utilize digital technologies as equalizers to challenge traditional power dynamics. This aligns with the “revenge of the nerds” hypothesis (30), which suggests that technology can provide traditionally less powerful individuals with new means of retaliation. For instance, studies have shown that cyberbullying victims may use technological accessibility as coping mechanisms to counter perceived powerlessness (31, 32).

Research has identified revenge as both a reactive coping strategy and a primary motive for cyberbullying (33). Victims often view retaliation as a way to regain control or restore perceived justice. This pattern of retaliation can create cyclical behavior, where individuals with higher levels of trait anger are more prone to experiencing and acting on feelings of vengeance, potentially leading to escalating patterns of cyberbullying behavior (34). Therefore, while acknowledging that revenge is only one of several possible motivators, the current study hypothesizes that revenge motivation may serve as a significant mediator linking trait anger to cyberbullying perpetration (Hypothesis 1).

### 1.2 The moderating role of online disinhibition

The relationship between revenge motives and cyberbullying perpetration among adolescents is not uniform, and online disinhibition may play a crucial moderating role in this dynamic. Online disinhibition refers to a psychological state wherein individuals experience reduced constraints in cyberspace, leading to increased self-expression on digital platforms (35). For adolescents harboring revenge motives, the online environment may present a particularly tempting arena for engaging in cyberbullying behaviors, regardless of whether they choose to conceal their identity. The disinhibitory effects could lower impulse control, exacerbating the impact of revenge desires and behaviors that are often triggered during adolescence (36). This is particularly evident in the Chinese context, where despite mandatory real-name registration systems on social platforms, cyberbullying behaviors persist, demonstrating that factors beyond anonymity contribute to online disinhibition effects (37).

Online disinhibition can be categorized into two distinct types: toxic and benign (16). Toxic online disinhibition encompasses negative behaviors not typically observed offline, such as aggressive behaviors, harsh criticisms, and engagement in darker aspects of the Internet. Studies have found that higher levels of toxic disinhibition among students were associated with increased cyberbullying perpetration (38, 39). Conversely, benign disinhibition refers to behaviors that facilitate self-understanding, personal development, and conflict resolution (37). While no studies have explicitly examined benign disinhibition’s influence on cyberbullying perpetration, its promotion of enhanced self-reflection and perspective-taking may help individuals better regulate their emotional responses and consider the consequences of their actions.

In the Chinese context, investigating different types of online disinhibition is particularly important given the country’s distinct digital ecosystem. Despite reduced anonymity, online disinhibition effects may still occur through other mechanisms such as asynchronicity and perceived distance in online communications. This knowledge would be instrumental in developing targeted interventions that account for China’s specific digital context.

Based on previous research, we hypothesize that toxic online disinhibition may strengthen both the direct relationship between trait anger and cyberbullying perpetration, and the relationship between revenge motives and cyberbullying perpetration, while benign online disinhibition may weaken these relationships. These moderation effects can be explained through the Risk Enhancing Model and the Risk Buffer Model (40, 41). The Risk Enhancing Model suggests that toxic online disinhibition could amplify both the direct influence of trait anger and the influence of revenge motives on cyberbullying perpetration. Conversely, benign online disinhibition may serve as a protective factor by promoting emotional regulation and prosocial alternatives to aggression.

## 2 Present study

**Figure 1:**
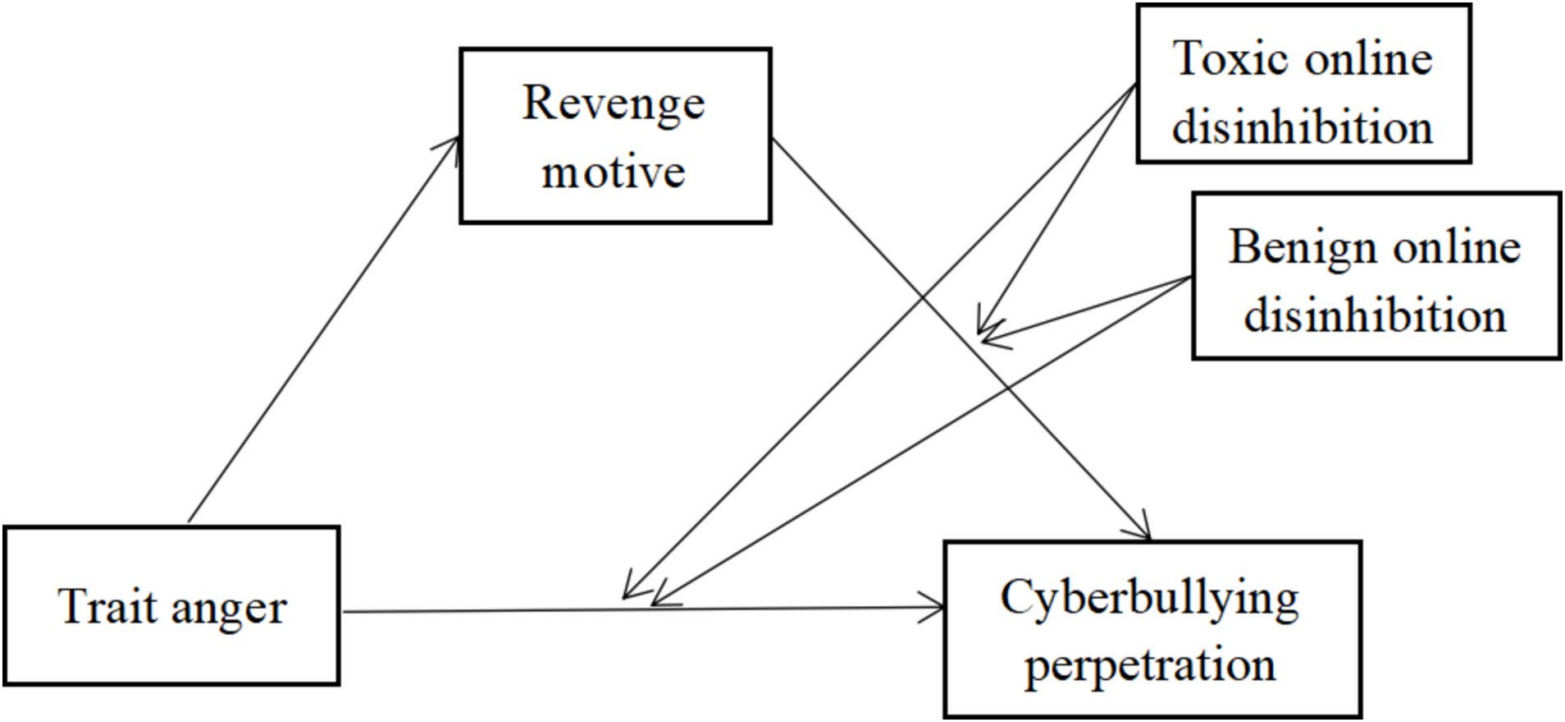
A moderated mediation model of trait anger, revenge motive, benign online disinhibition, toxic online disinhibition and cyberbullying perpetration.

The current study examines the relationships between trait anger, revenge motivation, online disinhibition, and cyberbullying perpetration among Chinese adolescents through a moderated mediation model (see Figure 1). We propose three hypotheses:

*Hypothesis 1*: Revenge motivation mediates the relationship between trait anger and cyberbullying perpetration.

*Hypothesis 2*: The relationship between trait anger and cyberbullying perpetration is moderated by online disinhibition, with stronger effects under high toxic disinhibition and weaker effects under high benign disinhibition.

*Hypothesis 3*: The relationship between revenge motivation and cyberbullying perpetration is moderated by online disinhibition, following the same pattern as Hypothesis 2.

## 3 Method

### 3.1 Participants and procedure

The study sample consisted of 1,574 students (males = 849; females = 725) recruited through convenience sampling from seven junior and senior high schools in Fujian Province. Participants included 725 females (46.1%) and 794 junior high school students (50.4%), with a mean age of 16.89 years (SD = 0.34, range: 13–19 years).

The grade distribution included 251 (31.6%) 7th graders, 250 (31.4%) 8th graders, 293 (37.0%) 9th graders, 301 (38.6%) 10th graders, 245 (31.4%) 11th graders, and 234 (30.0%) 12th graders (see Table 1 for additional demographic information). Data collection was conducted using an online questionnaire system, Wenjuanxing,^1^ from October to December 2023. Inclusion criteria for the study were having experience using the internet and a willingness to participate in the study. The exclusion criteria were if more than half of the items were answered in a repetitive fashion or if questionnaires were nonconforming (completion time less than 100 s). Verbal consent was obtained from all participants and their guardians prior to providing them with an online survey link containing the consent form and scales. As an incentive, participants who completed the survey were entered into a draw for an online prize. The study protocol was approved by the Ethics Committee of the authors’ university.

**Table 1:** Demographic characteristics of the participants (*n* = 1,574).

	*n* (%)
Gender	
Male	725 (46.1)
Female	849 (53.9)
Grade	
Grade 7 (age 13 ~ 14)	251 (31.6)
Grade 8 (age 14 ~ 15)	250 (31.4)
Grade 9 (age 15 ~ 16)	293 (37.0)
Grade 10 (age 16 ~ 17)	301 (38.6)
Grade 11 (age 17 ~ 18)	245 (31.4)
Grade 12 (age 18 ~ 19)	234 (30.0)
Online frequency	
Less than 2 h per day	869 (55.2)
2–5 h per day	571 (36.3)
5–8 h per day	97 (6.1)
More than 8 h per day	37 (2.4)
Family structure	
Complete family	1,414 (89.8)
Divorced family	114 (7.3)
Combined family	46 (2.9)

### 3.2 Measures

#### 3.2.1 Trait anger scale

Trait anger was assessed using the Chinese version of the Trait Anger Scale (42), adapted by Luo et al. (43). This 10-item scale employs a four-point response scale ranging from 1 (‘never’) to 4 (‘always’), with higher scores indicating higher levels of trait anger. A sample item is “When facing setbacks, I want to hit people.” The scale has demonstrated good validity and reliability among Chinese populations (43), with a Cronbach’s alpha of 0.89 and McDonald’s omega of 0.91 in the present study.

#### 3.2.2 Revenge motive scale

Revenge motivation was measured using the revenge motivation subscale of the Transgression-Related Interpersonal Motivations Inventory (44). This subscale consists of five items rated on a five-point Likert scale ranging from 1 (“strongly disagree”) to 5 (“strongly agree”), with higher scores indicating greater revenge motivation. A sample item is “I hope they can receive the deserved retribution.” This scale has shown adequate reliability and validity in China (45), with a Cronbach’s alpha of 0.95 and McDonald’s omega of 0.92 in the current study.

#### 3.2.3 Online disinhibition scale

The Online Disinhibition Scale (39) comprises 11 statements addressing two categories: benign online disinhibition (e.g., “I feel like a different person online”) and toxic online disinhibition (e.g., “Writing insulting things online is not bullying”). Participants rate their agreement on a 4-point Likert scale ranging from 1 (strongly disagree) to 4 (strongly agree), with higher scores indicating stronger online disinhibition. This scale has demonstrated adequate internal reliability and discriminant validity in Chinese populations (46). In this study, Cronbach’s alphas for the benign and toxic online disinhibition subscales were 0.88 and 0.83, respectively. The McDonald’s omega was 0.88 for both subscales.

#### 3.2.4 Cyberbullying perpetration scale

Cyberbullying perpetration was measured using the cyberbullying subscale of the Chinese version of the Cyber Bullying Inventory (47), revised by Chu and Fan (48). This 14-item scale assesses the frequency of cyberbullying perpetration over the past 3 months through information and verbal aggression on various online platforms. Responses range on a scale from 1 (never) to 4 (three times). A sample item is “Post an indecent photo of someone online without permission.” Item scores were averaged, with higher scores indicating higher levels of cyberbullying perpetration. This scale has demonstrated good reliability and validity in Chinese populations (48), with a Cronbach’s alpha of 0.86 and McDonald’s omega of 0.89 in the present study.

### 3.3 Data analysis

To assess potential common method bias, we conducted Harman’s single-factor test (49). The analysis revealed that the first factor accounted for 24.46% of the total variance, which is below the 40% threshold suggested by Lee et al. (50), indicating that common method bias was not a significant concern in this study.

We began with descriptive statistics and calculated Spearman’s rank correlation coefficients (Spearman’s rho). This non-parametric correlation method was chosen because it does not assume normal distribution of the variables, making it particularly suitable for our potentially skewed data. To test the hypothesized moderated mediation model, we employed Hayes's (51) PROCESS macro for SPSS (Model 15) with 5,000 bootstrap samples and 95% bias-corrected confidence intervals. The bootstrapping approach was specifically selected because it does not require the assumption of normal distribution for indirect effects, making it robust for testing mediation effects in non-normally distributed data. This method enabled us to simultaneously examine both mediating and moderating effects within a single statistical model.

All analyses controlled for gender, grade level, family structure, and frequency of online use. These covariates were included based on previous research documenting their associations with trait anger, retaliatory behavior, and cyberbullying perpetration (8, 34, 52). By controlling for these variables, we aimed to isolate the unique effects of the primary variables of interest and enhance the robustness of the findings.

## 4 Results

### 4.1 Preliminary analysis

Descriptive statistics and correlations among study variables (trait anger, revenge motivation, benign online disinhibition, toxic online disinhibition, and cyberbullying perpetration) are presented in Table 2. Trait anger was positively correlated with revenge motivation, benign online disinhibition, toxic online disinhibition, and cyberbullying perpetration.

**Table 2:** Descriptive statistics and Spearman’s rho correlations of the variables.

Variable	M ± SD	1	2	3	4	5
1. Trait anger	1.70 ± 0.50	–				
2. Revenge Motive	2.16 ± 1.07	0.47^***^	–			
3. Benign online disinhibition	2.51 ± 0.75	0.28^***^	0.35^***^	–		
4. Toxic online disinhibition	1.30 ± 0.55	0.22^***^	0.27^***^	0.22^***^	–	
5. Cyberbullying perpetration	1.05 ± 0.20	0.26^***^	0.31^***^	0.17^***^	0.24^***^	–

*N* = 1,574. ^***^p < 0.001.

### 4.2 Testing for the proposed model

The results from PROCESS Model 15 are presented in Table 3. Mediation analyses revealed that trait anger significantly predicted cyberbullying perpetration (*β* = 0.10, SE = 0.02, *t* = 4.12, *p* < 0.001, R^2^ = 0.31, *F* = 76.52, *p* < 0.001; path c). Trait anger also positively predicted revenge motivation (*β* = 0.45, SE = 0.02, *t* = 19.48, *p* < 0.001, R^2^ = 0.22, *F* = 90.40, *p* < 0.001; path a), and revenge motivation positively predicted cyberbullying perpetration (*β* = 0.11, SE = 0.02, *t* = 4.48, *p* < 0.001; path b). The indirect effect through revenge motivation was significant (*β* = 0.05, SE = 0.01, *p* < 0.001, 95% CI [0.02, 0.08]). These results support Hypothesis 1, indicating that revenge motivation partially mediated the relationship between trait anger and cyberbullying perpetration.

**Table 3:** Moderated mediation model testing the role of toxic online disinhibition in the relationship between trait anger and cyberbullying perpetration.

Predictor	Revenge motive			Cyberbullying perpetration		
	β	SE	t	β	SE	t
Gender	0.02	0.05	0.53	−0.09	0.04	−2.10^*^
Grade	0.02	0.01	1.57	−0.04	0.01	−2.54^*^
Family structure	0.10	0.05	2.05^*^	0.04	0.05	0.82
The time of online	0.08	0.03	2.35^*^	0.16	0.03	5.09^***^
Trait anger	0.45	0.02	19.48^***^	0.10	0.02	4.11^***^
Revenge motive				0.11	0.02	4.47^***^
toxic online disinhibition				0.11	0.02	4.61^***^
Revenge motive × toxic online disinhibition				0.22	0.02	11.11^***^
Trait anger × toxic online disinhibition				0.11	0.02	4.89^***^
*R* ^2^	0.22			0.31		
*F*	90.40^***^			76.53		

^***^p < 0.001, ^**^p < 0.01, and ^*^p < 0.05.

#### 4.2.1 Effects of toxic online disinhibition as a moderator

**Figure 2:**
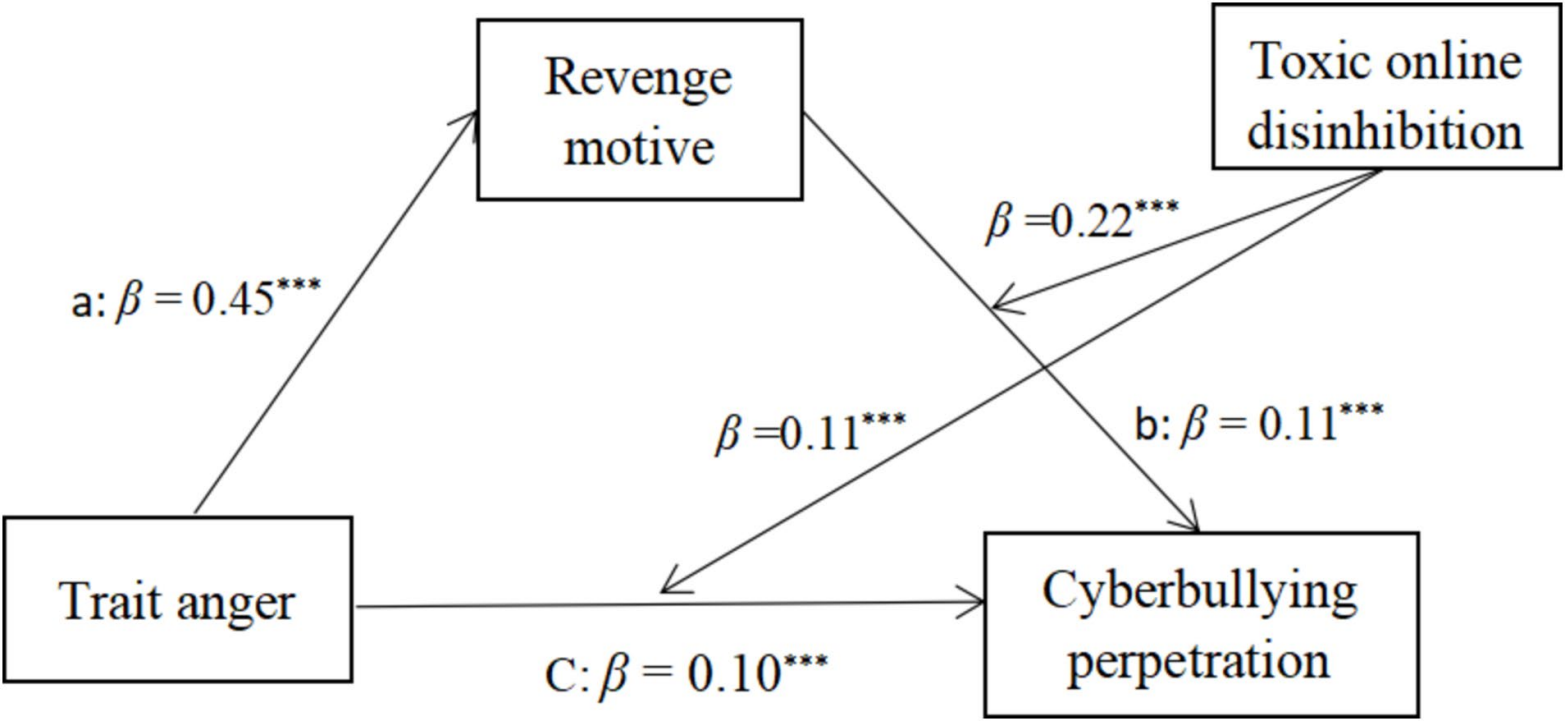
Final mode of the relationship between trait anger and cyberbullying perpetration.

Additionally, as shown in Table 3, toxic online disinhibition significantly moderated both the direct relationship between trait anger and cyberbullying perpetration (*β* = 0.11, *p* < 0.001) and the relationship between revenge motivation and cyberbullying perpetration (*β* = 0.22, *p* < 0.001). Figure 2 illustrates the moderated mediation pathways with standardized path coefficients.

**Figure 3:**
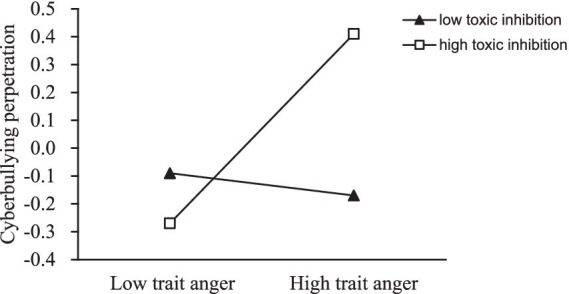
The relationship between trait anger and cyberbullying perpetration at two levels of toxic online disinhibition: (1) low toxic online disinhibition (1SD below the mean) and (2) high toxic online disinhibition (1SD above the mean).

To further examine the moderating role of toxic online disinhibition in the relationship between trait anger and cyberbullying perpetration, we conducted simple slope analyses. The relationship between trait anger and cyberbullying perpetration was plotted (Figure 3) for participants with low (1 SD below the mean) and high (1 SD above the mean) toxic online disinhibition. Simple slope analyses revealed that for individuals with low toxic online disinhibition, trait anger was not significantly associated with cyberbullying perpetration (*β* = −0.02, SE = 0.02, *t* = −0.67, *p* = 0.51). However, for individuals with high toxic online disinhibition, there was a significant positive association between trait anger and cyberbullying perpetration (*β* = 0.32, SE = 0.03, *t* = 11.03, *p* < 0.001).

**Figure 4:**
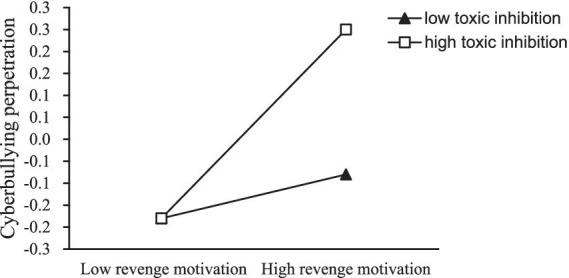
The relationship between revenge motive and cyberbullying perpetration at two levels of toxic online disinhibition: (1) low toxic online disinhibition (1SD below the mean) and (2) high toxic online disinhibition (1SD above the mean).

Similarly, we examined the moderating role of toxic online disinhibition in the relationship between revenge motivation and cyberbullying perpetration. The relationship was plotted (Figure 4) for participants with low and high toxic online disinhibition (±1 SD from the mean). Simple slope analyses indicated that for individuals with low toxic online disinhibition, the association between revenge motivation and cyberbullying perpetration was not significant (*β* = 0.05, SE = 0.03, *t* = 1.76, *p* = 0.07). In contrast, for those with high toxic online disinhibition, revenge motivation was positively associated with cyberbullying perpetration (*β* = 0.21, SE = 0.03, *t* = 6.87, *p* < 0.001).

The moderated mediation effect was further validated through bias-corrected percentile bootstrap analyses. Results confirmed that the indirect effect of trait anger on cyberbullying perpetration through revenge motivation was moderated by toxic online disinhibition. Specifically, for individuals with high toxic online disinhibition, there was a significant indirect effect of trait anger on cyberbullying perpetration via revenge motivation (indirect effect = 0.10, SE = 0.02, 95% CI [0.04, 0.15]). However, this indirect effect was not significant for individuals with low toxic online disinhibition (indirect effect = 0.02, SE = 0.01, 95% CI [−0.01, 0.05]). The index of moderated mediation was 0.05 (SE = 0.02, 95% CI [0.01, 0.09]). These findings provide partial support for Hypotheses 2 and 3.

### 4.3 Moderating effects of benign online disinhibition

Additionally, benign online disinhibition did not significantly moderate either relationship in the model. Specifically, the interaction between revenge motivation and benign online disinhibition was not significantly related to cyberbullying perpetration (*β* = 0.03, *t* = 0.93, *p* = 0.35, 95% CI [−0.03, 0.08]). Similarly, the interaction between trait anger and benign online disinhibition was not significantly associated with cyberbullying perpetration (*β* = 0.02, *t* = 0.89, *p* = 0.38, 95% CI [−0.03, 0.07]). The index of moderated mediation was 0.01 (SE = 0.03, 95% CI [−0.04, 0.06]). These non-significant findings suggest that benign online disinhibition may not play a protective role in reducing the impact of trait anger or revenge motivation on cyberbullying perpetration among Chinese adolescents. This contrasts with the significant moderating effects found for toxic online disinhibition and warrants further investigation into the differential effects of these two types of online disinhibition.

## 5 Discussion

This study employed a moderated mediation model to investigate the mechanisms underlying the relationship between trait anger and cyberbullying perpetration among Chinese adolescents. The findings demonstrate the mediating role of revenge motivation and the moderating effect of online disinhibition in this relationship.

### 5.1 The mediating role of revenge motive

The results suggest that revenge motivation may play a role in the relationship between trait anger and online aggressive behaviors among Chinese adolescents, though these findings should be interpreted with considerable caution given several conceptual and methodological limitations. Our data indicated that the vast majority of participants reported no cyberbullying perpetration within the three-month timeframe (*M* = 1.05, SD = 0.20), which substantially constrains the generalizability of our findings. The pathways within our mediation model warrant nuanced examination within these constraints. The initial pathway (trait anger → revenge motivation) suggests a potential relationship between trait anger and revenge-oriented cognitions, consistent with the cognitive model of anger (53) and previous research (34, 54). Importantly, since our study did not specifically sample cyberbullying victims or assess power dynamics between perpetrators and targets, the interpretation of “revenge motivation” in our non-victimized sample raises questions about whether the observed behaviors truly constitute cyberbullying or rather represent reactive online aggression aimed at power redistribution.

### 5.2 The role of online disinhibition

Our findings revealed that toxic online disinhibition strengthened both the direct relationship between trait anger and cyberbullying perpetration, and the relationship between revenge motives and cyberbullying perpetration, even within China’s real-name system context. This suggests that toxic disinhibition’s facilitation of aggressive online behaviors operates through mechanisms beyond anonymity, such as reduced social presence and perceived distance from consequences (16).

The relationship between benign and toxic online disinhibition warrants careful consideration. Our correlation analysis revealed a positive association between benign and toxic online disinhibition (see Table 2), suggesting they may not operate as opposing forces in online behavior. The absence of significant moderating effects for benign online disinhibition aligns with its conceptual nature - characterized by increased self-disclosure and prosocial behaviors rather than aggressive acts (55). This suggests that future research should examine benign disinhibition’s role primarily in relation to victimization rather than perpetration.

These findings highlight the need to reconsider how we conceptualize and address online disinhibition in cyberbullying prevention, particularly in contexts with real-name systems. Rather than focusing solely on identity disclosure, interventions may be more effective if they target the psychological mechanisms underlying toxic disinhibition and help adolescents develop stronger online self-regulation skills.

### 5.3 Cultural considerations in cyberbullying research

Our study provides preliminary insights into how trait anger, revenge motivation, and online disinhibition operate among Chinese adolescents, though several important limitations must be acknowledged. The most concrete contribution of our study lies in our finding that toxic disinhibition’s moderating effects persist even within China’s real-name system, suggesting that the psychological mechanisms of online disinhibition may operate independently of formal identity verification requirements.

Future research would benefit from explicitly measuring cultural values, social norms, and collective beliefs to better understand their potential influence on cyberbullying behaviors in Chinese contexts. Such research could help clarify whether and how cultural factors might shape the relationships we observed between trait anger, revenge motivation, and online behavior patterns.

### 5.4 Limitations and practical implications

Several methodological limitations warrant consideration. Firstly, this study’s cross-sectional design limits our ability to establish causal relationships among variables. Secondly, our reliance on self-report measures introduces potential social desirability bias. Additionally, a critical limitation concerns the low base rate of reported cyberbullying behavior in our sample, creating a potential floor effect that significantly impacts our findings’ interpretation. This clustering of responses at the lowest possible score raises questions about whether the observed relationships accurately reflect the true associations between variables. Future studies should examine these relationships among samples with documented cyberbullying behavior and incorporate both perpetrators and victims to better understand these associations.

Notwithstanding these limitations, our findings offer valuable insights for prevention and intervention strategies. Our results elucidate the pathways through which trait anger is associated with cyberbullying perpetration, though the practical significance of these relationships requires careful consideration given the extremely low base rate of reported behaviors. Additionally, our findings indicate that the indirect effects of trait anger on cyberbullying perpetration become significant for individuals with high toxic online disinhibition, even within China’s real-name system context. This suggests that intervention strategies should address psychological mechanisms that enable toxic disinhibition, such as asynchronous communication and reduced social cues, which can decrease adolescents’ self-control regardless of their anonymity status.

## Data Availability

The raw data supporting the conclusions of this article will be made available by the authors, without undue reservation.

## Footnotes

^1^ www.wjx.cn
